# Conversion surgery for stage IV gastric cancer after third-line immunotherapy: a case report

**DOI:** 10.3389/fonc.2024.1494669

**Published:** 2024-12-06

**Authors:** Sevindzh F. Evdokimova, Anna L. Kornietskaya, Larisa V. Bolotina, Iliya V. Kolobayev, Alexander A. Fedenko, Andrey D. Kaprin

**Affiliations:** ^1^ P. Hertsen Moscow Oncology Research Institute – Branch of the National Medical Research Radiological Centre of the Ministry of Health of the Russian Federation, Moscow, Russia; ^2^ Peoples’ Friendship University of Russia, Moscow, Russia

**Keywords:** gastric cancer (GC), gastroesophageal junction (GEJ), immunotherapy, conversion surgery, pembrolizumab, case report

## Abstract

The 5-year overall survival rate for stage IV gastric cancer is lower than 10%, despite the development of systemic therapy. Conversion surgery has shown to improve survival outcomes in patients with durable clinical response on chemotherapy. We report a clinical case of a patient, who underwent conversion surgery after pembrolizumab in the third-line setting for stage IV gastric cancer. The patient did not have recurrence for 22 months after conversion surgery.

## 1 Introduction

An estimated 26,500 cases of gastric cancer (GC) were diagnosed in the United States in 2023 (1). The incidence of GC has decreased in the Russian Federation, where it ranks fifth most commonly diagnosed cancer among the male population and ninth among the female population in 2022. However, GC still has a high mortality rate, ranking second in terms of mortality. The average age of patients with a first-time diagnosis of GC is 67.7 years. In addition, gastric and gastroesophageal junction adenocarcinoma demonstrates a high frequency of mortality: 5-year overall survival (OS) rate for patients with distant metastases accounts for 6%, according to the American Cancer Society (2, 3). Platinum-based chemotherapy is a gold standard of first-line treatment for metastatic GC with negative human epidermal growth factor receptor 2 (Her2-neu) expression and microsatellite stable subtype (MSS) (4–7). Real-world data show that 25% of patients with advanced gastric or GEJ cancer do not receive any treatment (8). Of those patients who are treated, 42% receive second-line therapy and only 19% have third-line therapy (8, 9). In the context of third-line therapy for GC, the KEYNOTE-012 trial assessed the efficacy of immune checkpoint inhibitors (ICIs), particularly pembrolizumab, in patients with PD-L1-positive recurrent or metastatic GC or GEJ cancer (GEJC). The results demonstrated significant anti-tumor activity and a manageable safety profile for pembrolizumab in patients with high PD-L1 expression (TPS ≥ 1). Following this, the KEYNOTE-059 trial further explored pembrolizumab as a third-line treatment for GC or GEJC patients, reporting an overall response rate (ORR) of 22.7% in patients with a combined positive score (CPS) ≥ 1, compared to 8.6% in those with PD-L1-negative tumors. Based on the favorable outcomes from KEYNOTE-059, the FDA granted accelerated approval for pembrolizumab for patients with recurrent, locally advanced, or metastatic GC or GEJC with CPS ≥ 1.

Conversion surgery is a preferred treatment option for patients with major clinical responses after first-line chemotherapy (10–13). Studies show that such patients can achieve significant improvement in survival outcomes; however, there has been a paucity of literature regarding the role of conversion surgery after second- or third-line systemic treatment for advanced GC. Furthermore, there is no standard approach after curative gastrectomy for M1 patients with a complete response of metastases after induction therapy.

We describe a patient with metastatic GC who underwent curative gastrectomy after a complete response of liver metastases on third-line pembrolizumab with no evidence of the disease for 22 months after surgery.

## 2 Case report

A 67-year-old man was admitted to the local hospital with abdominal pain in December 2019. Esophagogastroduodenoscopy (EGD) revealed infiltration of ulcerative changes in the antrum of the stomach. The pathological examination of the biopsied specimen indicated a poorly differentiated adenocarcinoma. Immunohistochemistry was negative for Her2neu, positive for programmed cell death ligand one expression (PD-L1) combined positive score=1% by clone 22C3, MSS. Computed tomography (CT) scan showed tumor invasion into the pancreas, multiple altered lymph nodes of the small omentum, gastrointestinal ligament, and carcinomatosis. The clinical diagnosis was cT4aN2M1 (per), Stage IV.

**Figure 1:**
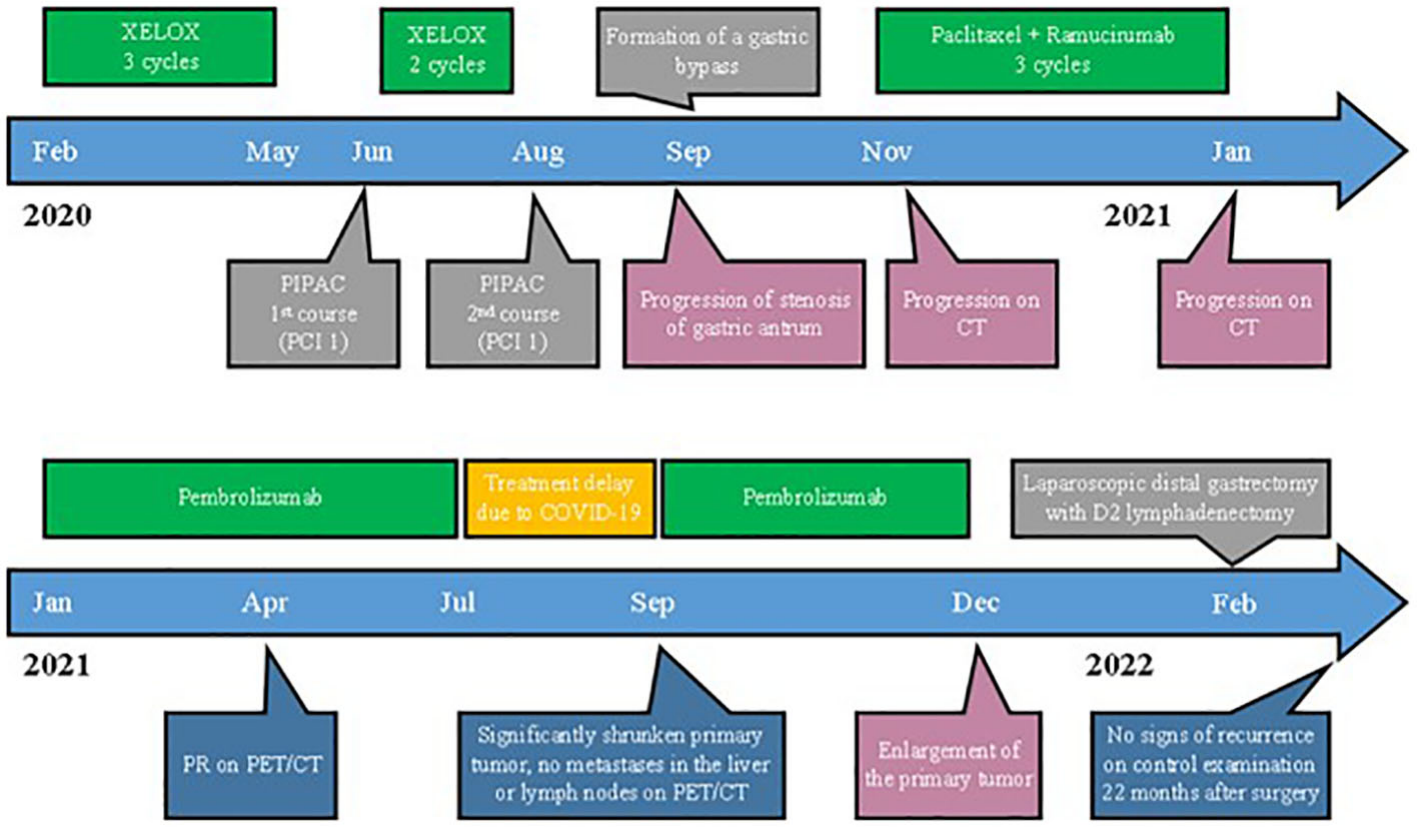
Timeline of the treatment and disease characteristics. PIPAC, pressurized intraperitoneal aerosol chemotherapy; PCI, peritoneal cancer index; CT, computed tomography.

The patient received first-line chemotherapy with XELOX between February 2020 and May 2020 (**Figure 1**). CT scan showed a slight reduction in tumor size, and in June 2020, the patient had the first session of pressurized intraperitoneal aerosol chemotherapy (PIPAC). The peritoneal cancer index (PCI) score was 1. Then two cycles of XELOX were administered, followed by the second course of PIPAC in August 2020. PCI score remained 1. After 1 month, the patient underwent a gastroenterostomy for pyloric stenosis.

In November of 2020, a CT scan of the abdomen showed progression of infiltrative changes in the stomach and enlarged perigastric and peripancreatic lymph nodes, and two metastases appeared in the liver.

**Figure 2:**
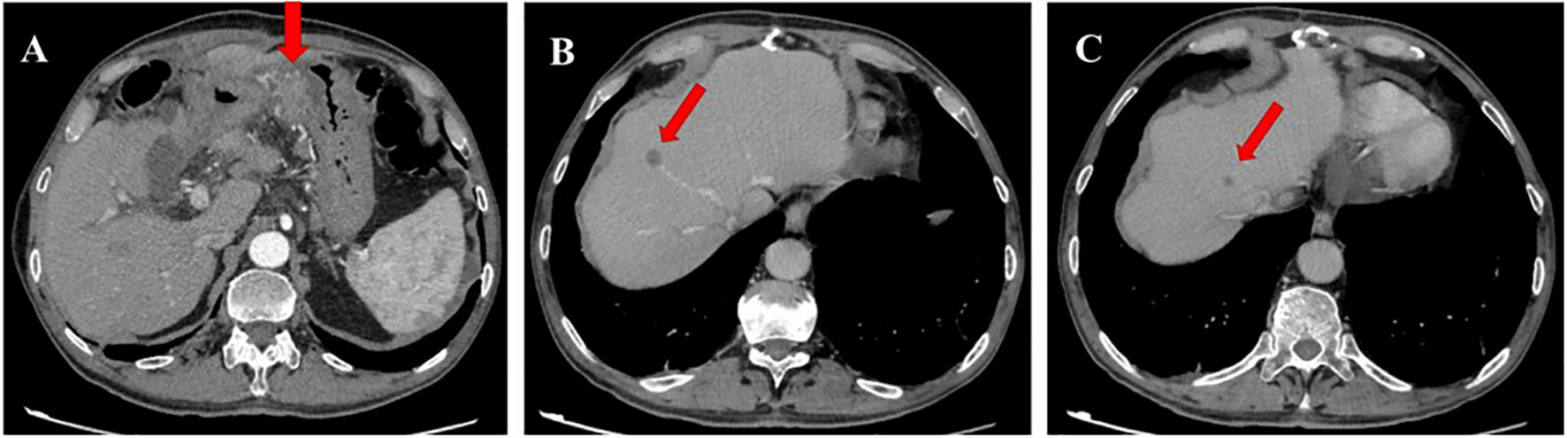
Contrast-enhanced computed tomography (CT) scan image in January 2021. **(A)** CT scan shows progression of infiltrative changes in the stomach. **(B, C)** CT scan shows new metastatic foces in the liver.

Therefore, the systemic therapy was switched to a combination of paclitaxel and ramucirumab, which is the standard second-line treatment for metastatic GC. After three cycles, a CT scan showed growth of existing metastases and the appearance of new lesions (**Figure 2**).

Hence, in January 2021, the patient started the third-line pembrolizumab due to positive CPS (1%).

**Figure 3:**
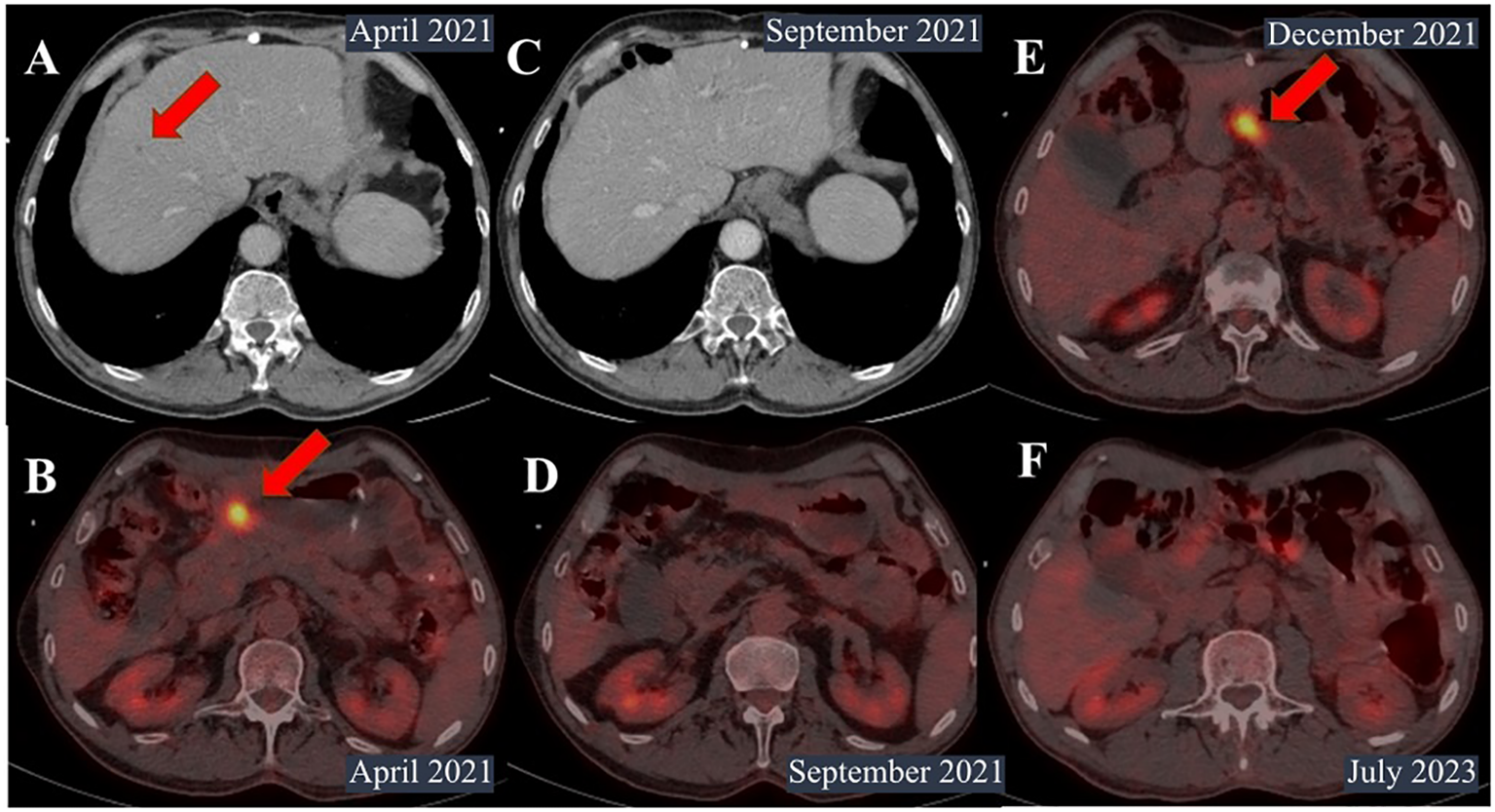
Contrast-enhanced computed tomography (CT) and PET scan images. **(A)** CT scan in April 2021. The size of metastatic foci in the S8 segment of the liver decreased from 11 mm to 8 mm. **(B)** Positron emission tomography (PET) in April 2021 showed a decrease in the size of infiltration in the distal part of the stomach from 73 mm × 104 mm to 39 mm × 28 mm. **(C, D)** PET scan in September 2021. No metastases in the liver or lymph nodes were identified. **(E)** PET scan in December 2021. The primary tumor was noted in the small curvature of the pylorus up to 18 mm × 24 mm with hypermetabolism of fluorodeoxyglucose (SUVmax=11.29). **(F)** PET scan in July 2023. No signs of recurrence were noted.

Positron emission tomography (PET) in April 2021 showed a decrease in the size of infiltration in the distal part of the stomach from 73 mm × 104 mm to 39 mm × 28 mm and perigastric and peripancreatic lymph nodes from 20 mm to 7 mm. One metastatic lesion in the liver disappeared, and another decreased from 11 mm to 8 mm with no abnormal uptake (**Figures 3A, B**).

However, the patient was infected with coronavirus disease and had a break in treatment for 2 months between July and September 2021. PET scan showed a significantly decrease in primary tumor, with no metastasis in the liver or lymph nodes (**Figures 3C, D**).

The patient continued pembrolizumab until December 2021, when enlargement of the primary tumor and the appearance of hypermetabolism of fluorodeoxyglucose were noted (**Figure 3Е**).

Due to the absence of distant metastases, the patient underwent conversion surgery with laparoscopic distal gastrectomy with D2 lymphadenectomy (previously done staging laparoscopy indicated no peritoneal dissemination and negative peritoneal cytology). The postoperative histology showed well-differentiated adenocarcinoma, 25 mm tumor node with muscle layer invasion, and clear lymph nodes.

Since then, the patient has not received any treatment and showed no signs of recurrence on follow-up examinations with PET and EGD every 3 months for 22 months after surgery (**Figure 3F**).

## 3 Discussion

Advanced GC with peritoneal or distant metastases has a poor prognosis, despite the development of targeted drugs or immune checkpoint inhibitors; the median overall survival (OS) does not reach 1 year (8, 14–17). Although stage IV GC can present in various tumor characteristics and biology (18–20), in patients with major clinical response to systemic therapy, CS could provide a promising option and prolong their survival outcomes (21–23). It is important to convert the patient in time because systemic treatment could result in chemoresistance or cause severe adverse effects.

However, there is necessity to determine prognostic markers of patients, who benefit from CS.

The multicenter retrospective study by Kano et al. analyzed four cohorts of patients (n=79). Patients from the first cohort had resectable GC if they had positive peritoneal cytology, para-aortic lymph node metastases, or solitary liver metastasis minor 5 cm, and patients from other cohorts had unresectable disease (24, 25). Resectable GC (HR, 0.378; 95% CI, 0.173–0.824; p = 0.014) and R0 resection (HR, 0.439; 95% CI, 0.227–0.847; p = 0.014) of all metastatic sites were significant prognostic markers of favorable OS by multivariate Cox regression analysis.

In addition, Lin Ni et al. showed that the prognostic marker GPR176 correlates with sensitivity to drug therapy in GC (25). They found that high expression of GPR176 in tumors is associated with poor prognosis. Moreover, both CTLA4- and PD-1 positive and negative gastric adenocarcinomas with low expression of GPR176 had better responses to ICIs.

We report a clinical case of a patient, who progressed on two lines of systemic therapy; however, he had a durable response on pembrolizumab with MSS and low PD-L1 status, and subsequent conversion surgery. The described above clinical case is the fifth case report of CS after the third-line immunotherapy, and in the majority of these reports, no adjuvant therapy was administered. In addition, in the present clinical case, the patient did not receive adjuvant therapy, as there is currently limited evidence supporting its use. For instance, a retrospective study conducted at the National Cancer Center in China evaluated 122 patients who underwent conversion surgery (26). Of these, only 80 patients (65.6%) received postoperative adjuvant chemotherapy—either S-1 alone (n=36) or S-1 combined with platinum (n=28). The treatment was administered for a median of three cycles. Regarding OS, no significant difference was observed between the adjuvant therapy group and the observation group (63.9 months vs. 50.5 months, p=0.72). However, the adjuvant group did show a survival benefit in progression-free survival (PFS), with a median of 29.7 months compared to 14.6 months in the observation group (p=0.009). In a clinical case reported by Kosuke Fukuda et al., a patient underwent conversion surgery after second-line treatment with paclitaxel in combination with ramucirumab, followed by postoperative adjuvant chemotherapy with S-1 for 6 months (27). The patient survived without recurrence for 42 months after conversion surgery. In another case by Ryu Matsumoto et al., involving conversion surgery after third-line treatment with nivolumab, despite achieving a complete pathological response, the patient continued adjuvant therapy with nivolumab (28). Given the radical R0 surgical resection, the well-differentiated adenocarcinoma of the primary tumor, and the fact that the role of adjuvant immunotherapy remains unclear with limited supporting evidence, the decision in our case was made to proceed with active surveillance.

In the present case, given that primary tumor enlarged, but disappearance of distant metastases was shown after 11 months of immunotherapy, conversion surgery was performed. No peritoneal dissemination or positive peritoneal cytology was observed via staging laparoscopy.

This patient had favorable prognostic factors, such as a durable response on treatment, no peritoneal dissemination or positive peritoneal cytology observed via staging laparoscopy, and R0 resection of the primary tumor. Obviously, CS for stage IV GC is not routinely performed due to low objective responses on chemotherapy, especially after third-line treatment, because of tumor aggressiveness, performance status of the patient, and unresectable metastases. Considering that CS can improve survival outcomes, it is crucial to select patients based on favorable prognostic factors, such as a durable response to systemic treatment, limited extent of metastatic disease, negative peritoneal metastasis and negative peritoneal cytology findings, good performance status, and the potential for achieving complete resection. A multidisciplinary approach is essential for careful patient selection, maximizing the likelihood of successful outcomes and improving overall survival.

## 4 Conclusion

This case report illustrates the long-term survival for 22 months of the patient after CS for advanced GC without adjuvant therapy. Conversion surgery could be a treatment option after third-line immunotherapy; however, there is no standard treatment approach for postoperative treatment after conversion surgery for stage IV GC. Further research is needed to identify predictive biomarkers of response to immune checkpoint inhibitors and determine a cohort of patients, who will benefit from subsequent surgery.

## Data Availability

The raw data supporting the conclusions of this article will be made available by the authors, without undue reservation.
